# Characterization of lung adenocarcinoma based on immunophenotyping and constructing an immune scoring model to predict prognosis

**DOI:** 10.3389/fphar.2022.1081244

**Published:** 2022-12-19

**Authors:** Mengfeng Liu, Qifan Xiao, Xiran Yu, Yujie Zhao, Changfa Qu

**Affiliations:** ^1^ Department of Thoracic Surgery, Harbin Medical University Cancer Hospital, Harbin Medical Sciences University, Harbin, China; ^2^ Regional Marketing Department, YuceBio Technology Co., Shenzhen, China

**Keywords:** LUAD, immune, immunophenotyping, immune scoring model, prognosis

## Abstract

**Background:** Lung cancer poses great threat to human health, and lung adenocarcinoma (LUAD) is the main subtype. Immunotherapy has become first line therapy for LUAD. However, the pathogenic mechanism of LUAD is still unclear.

**Methods:** We scored immune-related pathways in LUAD patients using single sample gene set enrichment analysis (ssGSEA) algorithm, and further identified distinct immune-related subtypes through consistent clustering analysis. Next, immune signatures, Kaplan-Meier survival analysis, copy number variation (CNV) analysis, gene methylation analysis, mutational analysis were used to reveal differences between subtypes. pRRophetic method was used to predict the response to chemotherapeutic drugs (half maximal inhibitory concentration). Then, weighted gene co-expression network analysis (WGCNA) was performed to screen hub genes. Significantly, we built an immune score (IMscore) model to predict prognosis of LUAD.

**Results:** Consensus clustering analysis identified three LUAD subtypes, namely immune-Enrich subtype (Immune-E), stromal-Enrich subtype (Stromal-E) and immune-Deprived subtype (Immune-D). Stromal-E subtype had a better prognosis, as shown by Kaplan-Meier survival analysis. Higher tumor purity and lower immune cell scores were found in the Immune-D subtype. CNV analysis showed that homologous recombination deficiency was lower in Stromal-E and higher in Immune-D. Likewise, mutational analysis found that the Stromal-E subtype had a lower mutation frequency in TP53 mutations. Difference in gene methylation (ZEB2, TWIST1, CDH2, CDH1 and CLDN1) among three subtypes was also observed. Moreover, Immune-E was more sensitive to traditional chemotherapy drugs Cisplatin, Sunitinib, Crizotinib, Dasatinib, Bortezomib, and Midostaurin in both the TCGA and GSE cohorts. Furthermore, a 6-gene signature was constructed to predicting prognosis, which performed better than TIDE score. The performance of IMscore model was successfully validated in three independent datasets and pan-cancer.

## 1 Introduction

Lung cancer still serves as the most common malignancy and the foremost cause of cancer death in the world (Thandra et al., 2021). Lung adenocarcinoma (LUAD) as a main subtype of lung cancer belong to the larger group of non-small cell lung cancer, and accounts for about 40% of all lung cancer cases Moreover, it is strongly associated with smoking (Pintarelli et al., 2019; Spella and Stathopoulos, 2021). Although treatment modalities such as surgery, chemoradiotherapy, targeted therapy, and immunotherapy have been widely used for the treatment of lung cancer, but the prognosis remains dismal (Ladanyi and Pao, 2008; Bronte et al., 2010; Denisenko et al., 2018), with a 5-year survival rate as low as less than 10% (Hirsch et al., 2017).

Cancer evolution is influenced by complex interactions between tumor cells and host immune responses within the tumor microenvironment (Taube et al., 2018). Different immune cell populations are actively involved in tumor immune microenvironment (TIME), however, their relationship is currently unclear in LUAD therapy (Zhang et al., 2020). Immune cells are both positive and negative regulators of cancer progression (Zamarron and Chen, 2011). For example, B-cells exert antitumor functions by enhancing T-cell immunity, stimulating the production of interferon-γ and helping natural killer (NK) cells against tumors (Sorrentino et al., 2011). However, B-cells also suppress immune responses and promote angiogenesis (Schwartz et al., 2016). Considering the importance of TIME in cancers, we attempted to classify LUAD based on immune pathways and compared their characteristics.

Disease prediction models have been widely used to evaluate patient survival and other prognostic indicators in LUAD (Jiang et al., 2020; Jiang et al., 2022). Recently, a novel model related to lactate metabolism for predicting overall survival and immune signature in LUAD was reported and different immune signatures were built (Jiang et al., 2022). Moreover, an increasing number of LUAD prognostic biomarkers have been discovered by analyzing expression profiles from public databases and related clinical information (Jiang et al., 2020).

Due to advances in genetic technology, various molecular subtypes and gene expression studies have been published and popularized for the identification of prognostic biomarkers (Li and Wang, 2021). However, the prognostic manifestations of established biomarkers are controversial and limited. In the current study, we identified and classified three immune subtypes and a six-gene combination capable of predicting survival in patients with LUAD.

## 2 Materials and methods

### 2.1 Data collection

To better understand the pathogenesis of LUAD, RNA-Seq data of LUAD with clinical survival and characteristic information were downloaded from TCGA database by using TCGA GDC API. Fragments per kilobase of transcript per million fragments mapped (FPKM) were converted into transcripts per million (TPM), and then Log2 was transformed for subsequent analysis. In addition, we downloaded the GSE37745, GSE50081, GSE30219, GSE31210 microarray datasets with survival times from the GEO database (Yamauchi et al., 2012; Botling et al., 2013; Rousseaux et al., 2013; Der et al., 2014). For GEO, MINiML formatted family file(s) were downloaded and samples was preprocessed using RMA implemented in affy package. Normalized data values were transformed in log2 space and used in subsequent analysis.

### 2.2 Data quality control

To ensure the accuracy of downstream analysis, the RNA-Seq data of TCGA-LUAD without clinical follow-up information or survival time were removed. Next, the data without status were filtered. Then, only the genes with more than one expression in more than 50% of the samples were retained.

For GSE data, firstly, normal tissues, samples without clinical follow-up information, samples without OS data and samples without status were filtered. Then, we converted the probes to symbols according to the annotation file.

### 2.3 Batch effect processing

The *removeBatchEffect* function of the limma (R package) was used to remove batch effects between different datasets (Ritchie et al., 2015). Principal component analysis (PCA) was used to observe the batch effect (Supplementary Figure S1).

### 2.4 LUAD classification based on pathway score

In order to explore the molecular typing of LUAD, single sample gene set enrichment analysis (ssGSEA) analysis was used to calculate the score related to immune pathway (Supplementary Table S1) (Gao et al., 2021). Next, ConsensusclusterPlus (R Bioconductor/R package) software was used for consensus clustering analysis with “pam” arithmetic and “pearson” distance, and the input was a sample matrix pool of immune pathway scores (Wilkerson and Hayes, 2010). Then, we determined the optimal number of clusters according to the cumulative distribution function (CDF).

### 2.5 Gene mutation analysis

The *mutect2* software was applied to perform gene mutation analysis (Benjamin et al., 2019). The tumor promoting genes were obtained from previous study. The fisher’s test was used to screen genes with significantly high frequency mutations in each subtype, with a threshold of *p-*value < 0.05. Moreover, the *maftools* software (R package) was used to calculate the tumor mutational burden (TMB) score (Mayakonda et al., 2018).

### 2.6 Copy number variation (CNV) analysis

GISTIC2.0 was used to analyze the change of CNV (Mermel et al., 2011). If the ratio was greater than 0.2, it was considered as Gain, if the ratio was less than −0.2, it was considered as Loss, and the rest was considered as Diploid.

### 2.7 Methylation analysis

The 450K methylation data of LUAD were used to perform methylation analysis of EMT-promoting genes (Wang and Zhou, 2013), and missing values were imputed using the KNN algorithm of the *impute* software (R package) (Hastie et al., 2011).

### 2.8 Treatment plan sensitivity analysis

We used the tumor immune dysfunction and exclusion (TIDE) (https://tide.dfci.harvard.edu/) algorithm to predict response of immunotherapy (Jiang et al., 2018). The higher the TIDE prediction score, the higher the likelihood of immune escape, and the lower the likelihood that the patient would benefit from immunotherapy. Moreover, IC50 (half maximal inhibitory concentration) analysis was used to determine the sensitivity of different subtypes to chemotherapy drugs using pRRophetic method.

### 2.9 Weighted gene co-expression network analysis (WGCNA)

WGCNA was performed by WGCNA (R package) to filter hub genes in the module related to different LUAD subtypes (Langfelder and Horvath, 2008). Hub genes refer to genes that play key roles in modules and are generally closely related to shape. The hub genes were further subjected to KEGG pathway analysis by using clusterProfiler (R Bioconductor/R package). *p-*value < 0.05 was considered significance.

### 2.10 IMscore predicted by prognostic model

IMScore model was constructed using univariate Cox regression analysis and LASSO analysis. Finally, six prognostic genes were obtained, including MARCKS, CDK2, SFN, SSBP1, MRE11 and FZD7. IMScore = ΣExp(i)*β(i), where i refers to the immune-related prognostic genes, Exp refers to the expression levels of genes, and β refers to the LASSO coefficients.

## 3 Results

### 3.1 LUAD classification based on pathway scores

**FIGURE 1:**
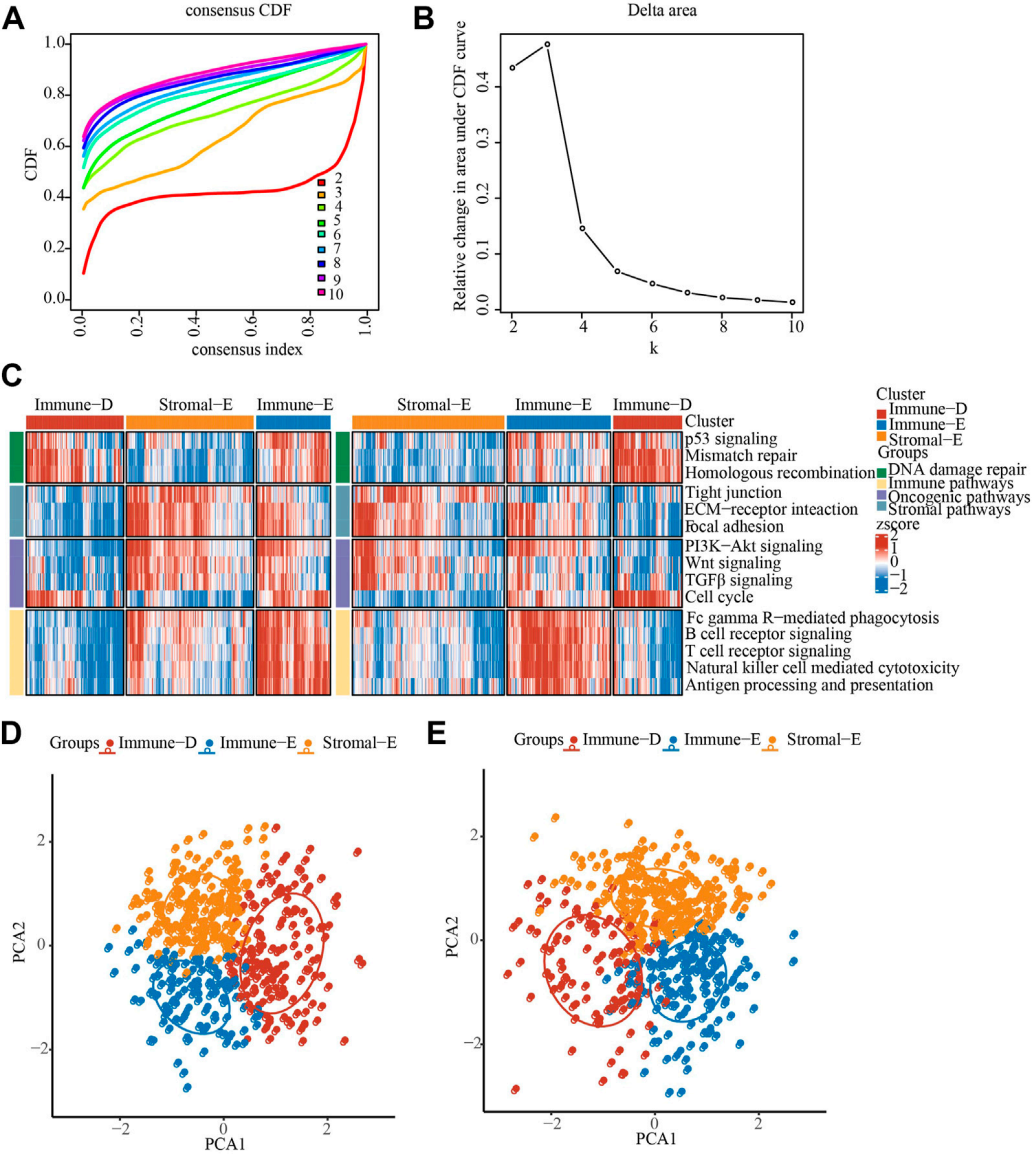
Identification of LUAD immunophenotyping. **(A)**, The cumulative distribution function (CDF) of different consensus index. **(B)**, The CDF Delta area curve. **(C)**, The heat map showing the immune pathway score in TCGA and GSE cohorts. **(D)**, PCA analysis in TCGA cohorts, different color means different subtypes. **(E)**, PCA analysis in GSE cohorts, different color means different subtypes.

The pathway scores of different samples in the TCGA and GSE cohorts were calculated by ssGSEA algorithm, and then consensus clustering analysis was performed on the pathway scores. The optimal number of clusters was determined according to the CDF, and the CDF Delta area curve was observed. When it was selected as 3, it has a relatively stable clustering result, and finally we choose k = 3 to obtain three related subtypes, including immune-Enrich subtype (Immune-E), stromal-Enrich subtype (Stromal-E) and immune-Deprived subtype (Immune-D) (Figures 1A,B). A heatmap showing the trends in pathway scores for each sample demonstrated a good discrimination between the three different subtypes (Figure 1C). In addition, PCA analysis between different subtypes showed that in both datasets, there were distinct boundaries between different subtypes (Figures 1D,E).

### 3.2 Analysis of clinical characteristics of different subtypes

Survival analysis showed a better prognosis in the Stromal-E subtype and a worse prognosis for the Immune-D subtype, which was consistent in the TCGA cohort (left) and the GSE cohort (right) (Supplementary Figure S2A). Analysis of differences in the distribution of clinical features among subtypes in the TCGA dataset showed significant differences in age, sex as well as the distribution of T Stage among subtypes (Supplementary Figure S2B). Comparative analysis of the molecular subtypes with the six previously identified immunophenotypes (Thorsson et al., 2018) showed that the previously published immunophenotypes were significantly different from our current study, for example, the Immune-E subtype had the highest proportion of the C2 subtype and C3 subtype had the best prognosis and also contributed the largest proportion in Stromal-E subtype. These results supported the reliability of our immunosubtyping (Supplementary Figure S2C)

### 3.3 Immune characteristics among different LUAD subtypes

**FIGURE 2:**
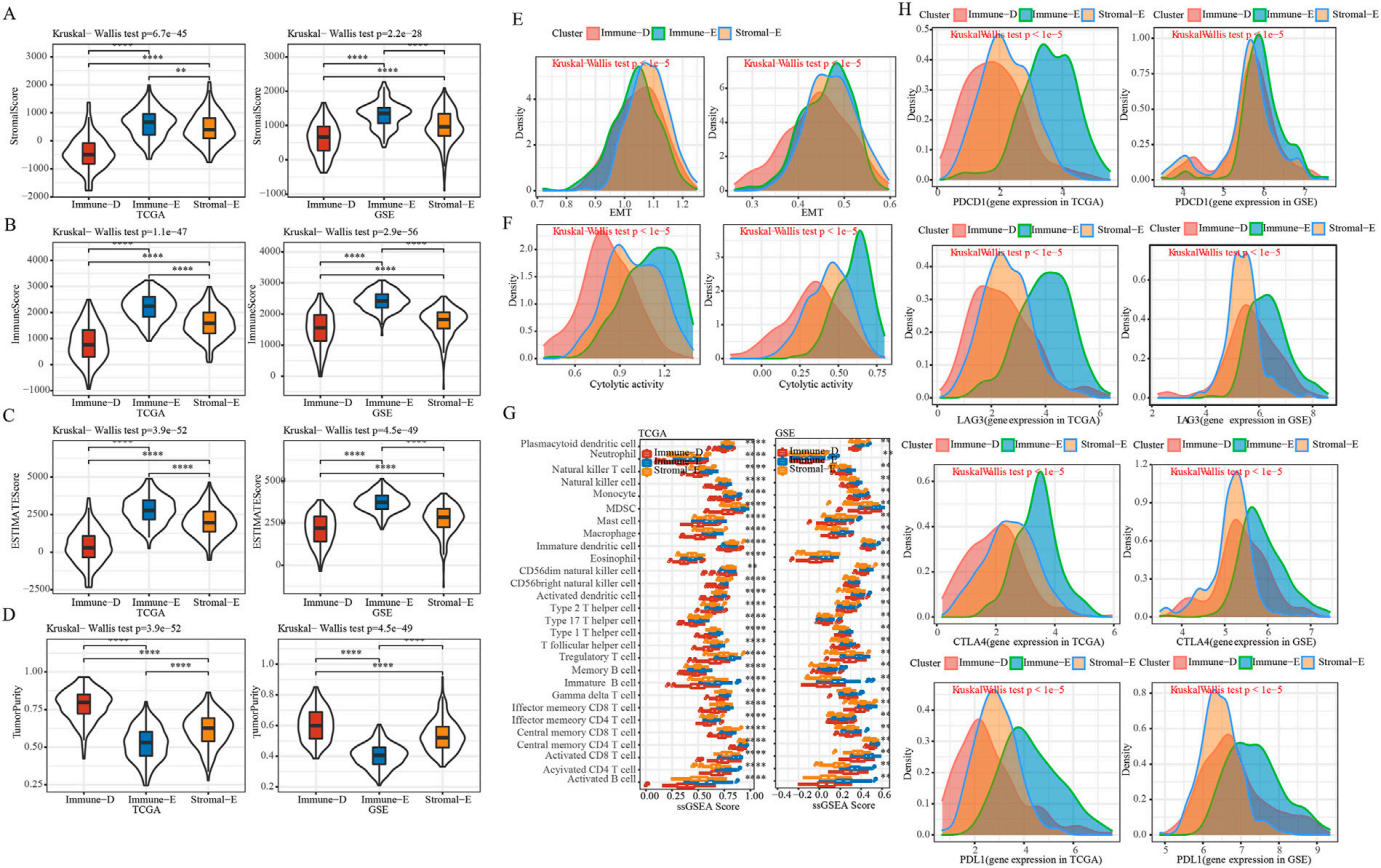
Immune features of different subtypes. **(A)**, Comparison of distribution of StromalScore in subtypes. **(B)**, Comparison of distribution of ImmuneScore in subtypes. **(C)**, Comparison of distribution of ESTIMATEScore in subtypes. **(D)**, Comparison of distribution of TumorPurity in subtypes. **(E)**, EMT score Comparison of distribution among subtypes. **(F)**, Comparison of distribution of Cytolytic activity scores among subtypes. **(G)**, Comparison of distribution of immune cell scores among subtypes. **(H)**, Density distribution map of gene PD1, CTLA4, LAG3 and PD-L1 in different subtypes. * means *p-*value < 0.05, ** means *p-*value < 0.01, *** means *p-*value < 0.001, **** means *p-*value < 0.0001.

In order to further understand the immune characteristics among the various subtypes of LUAD, we first used the *Estimate* software to evaluate the immune scores and tumor purity score of the TCGA and GSE cohorts. The results demonstrated that the immune score, stromal score, and ESTIMATE score were the highest in Immune-E subtype both in the TCGA and GSE cohorts (Figures 2A–C). The opposite was true for the tumor purity score (Figure 2D). Epithelial-mesenchymal Transition (EMT) is closely related to tumor metastasis and recurrence (Wang and Zhou, 2013). Therefore, we applied the ssGSEA algorithm to evaluate the difference in EMT scores between different subtypes. The results showed that in the TCGA cohort, the Stromal-E subtype had the highest EMT score, and in the GSE cohort Immune-E subtype was the highest (Figure 2E). Cytolytic activity is associated with immunotherapy, and here ssGSEA was used to assess differences in Cytolytic activity score (Rooney et al., 2015). We found that both in TCGA and GSE cohorts, the score was highest in Immune-D subtype and the lowest in Immune-E subtype (Figure 2F). To further understand the status of different immune cells, we assessed immune cell infiltration between different subtypes and found the highest scores in Immune-E, such as Plasmacytoid dendritic cell, Regulatory T cell and Gamma delta T cell. This was also the same in the TCGA and GSE cohorts (Figure 2G). Moreover, we determine the expression level of PD1, PD-L1, CTLA4 and LAG3, and observed that the expression level was higher in Immune-E subtype (Figure 2H).

### 3.4 Mutational analysis of tumor driver genes in different subtypes

**FIGURE 3:**
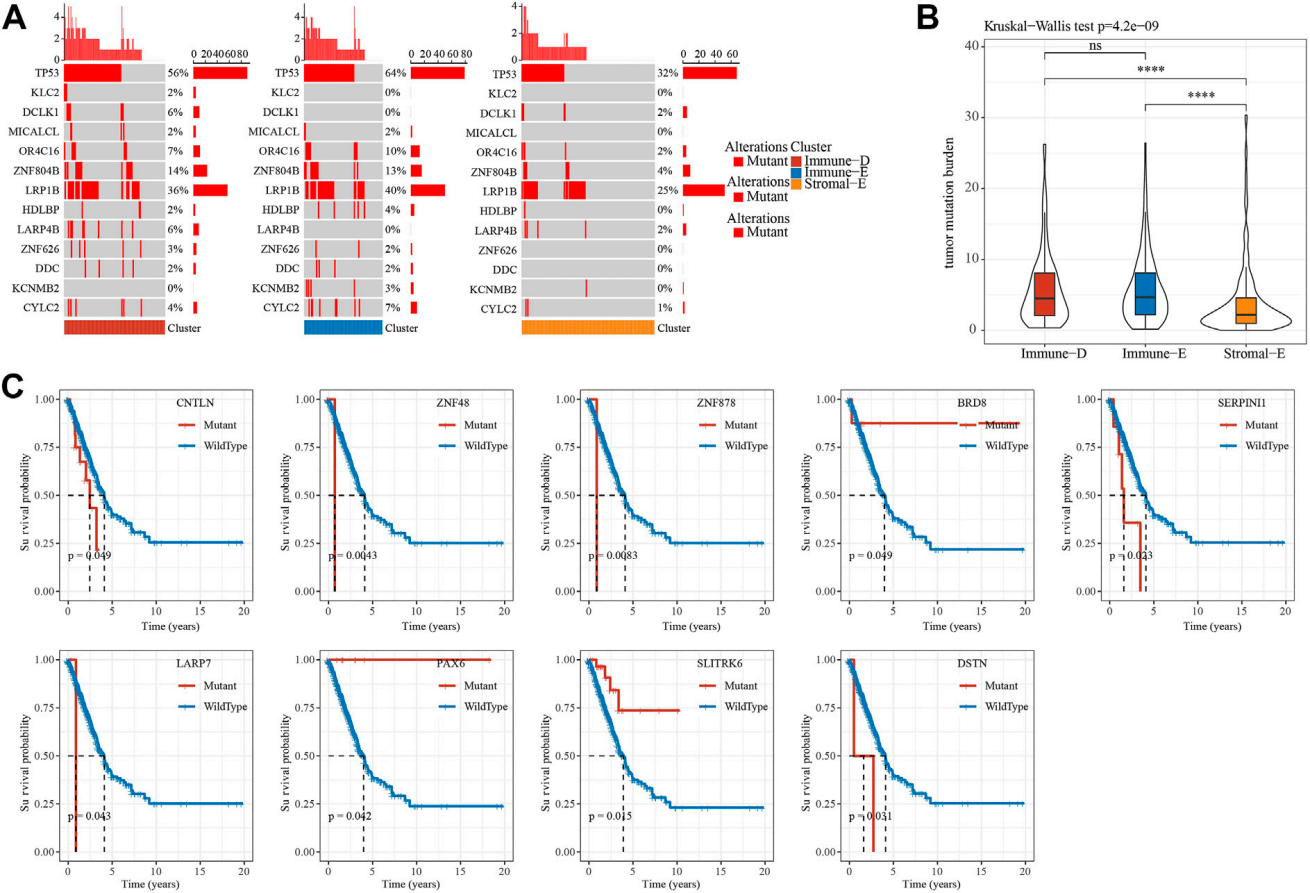
Mutation features of different subtypes. **(A)**, Waterfall plot of tumor mutated genes in different subtypes. **(B)**, Differential analysis of TMB distribution in different subtypes. **(C)**. Kaplan-Meier survival analysis of mutation and wild-type tumor driver genes. Ns means no significance, * means *p-*value < 0.05, ** means *p-*value < 0.01, *** means *p-*value < 0.001, **** means *p-*value < 0.0001.

A total of 172 tumor driver genes were obtained from previous study (Gao et al., 2013). Mutation analysis of tumor driver genes in different subtypes of TCGA dataset found that 13 genes had different mutations in different groups and TP53 had the highest mutation frequency in the Immune-E subtype (Figure 3A). The results of TMB analysis in different subtypes found that TMB in Stroma-E subtype was significantly lower than that in Immune-E and Immune-D subtypes. There was no difference in TMB distribution between Immune-E and Immune-D subtypes in TCGA dataset (Figure 3B). In addition, survival curve (KM) analysis of mutations in driver genes and wild-type samples in TCGA dataset demonstrated that mutations in nine genes were significantly different from wild-type, including CNTLN, ZNF48, ZNF878, BRDS, SERPINI1, LARP7, SLITRK6 and DSTN (Figure 3C). Those analysis indicated that three subtypes may predict mutation status.

### 3.5 CNV analysis of tumor driver genes in different subtypes

**FIGURE 4:**
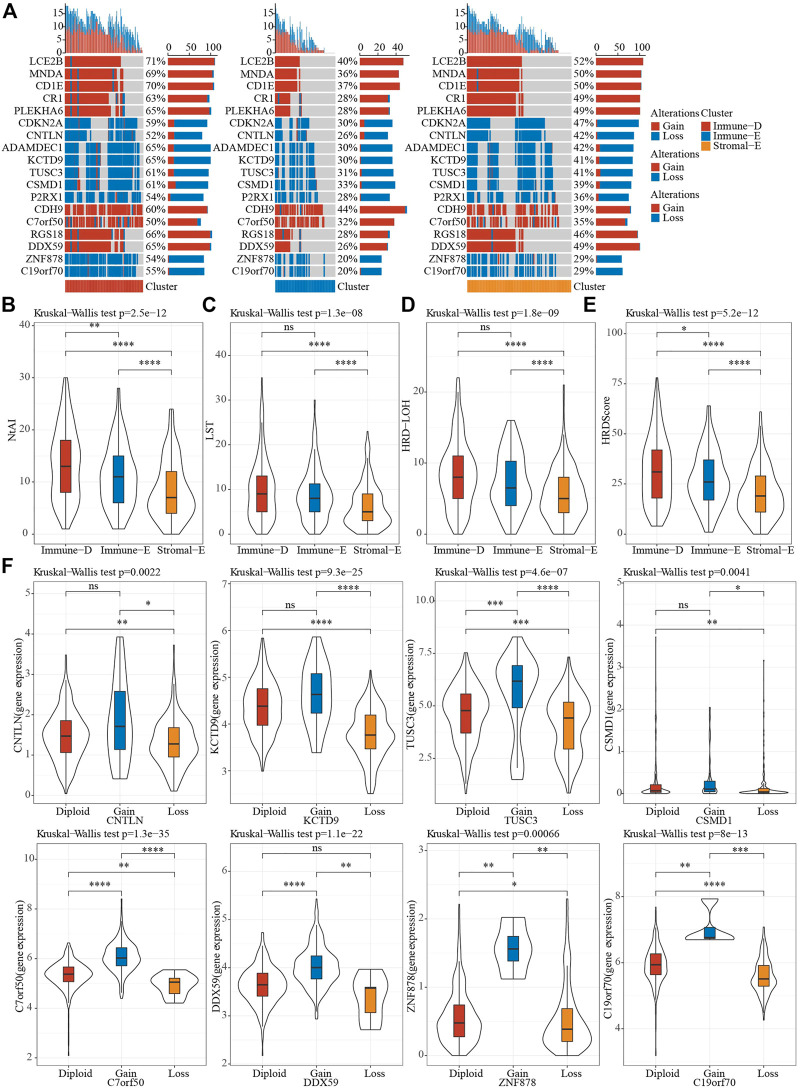
CNV features of different subtypes. **(A)**, CNV distribution of tumor driver genes. **(B–E)**, Differences in NtAI, LST, LOH and HRD score of different subtypes, respectively. **(F)**, Differences in gene expression by tumor driver gene CNV groupings. Ns means no significance, * means *p-*value < 0.05, ** means *p-*value < 0.01, *** means *p-*value < 0.001, **** means *p-*value < 0.0001.

A total of 159 of 172 tumor driver genes had CNV data. To understand CNV in tumor driver genes, GISTIC2 was used to perform CNV analysis. The results showed that the amplification and deletion of 159 driver genes were significant in different subtypes in TCGA dataset, and top 18 genes were visualized. Notably, immune-D had the largest amounts of CNVs (Figure 4A). Homologous recombination deficiency (HRD) is associated with a poorer cancer prognosis (Knijnenburg et al., 2018). Loss of heterozygosity (LOH), LST (large-scale state transitions), NtAI (number of telomeric allelic imbalances) score and HRD score were selected to assess the HRD status in different subtypes, and we found that the above scores were the lowest in Stromal-E and highest in Immune-D (Figures 4B–E). The expression level of tumor driver genes in the gene CNV grouping showed that the expression of the genes corresponding to the grouping with Gain was higher, while that corresponding to the grouping with Loss was lower (Figure 4F).

### 3.6 Methylation analysis of genes in different subtypes

**FIGURE 5:**
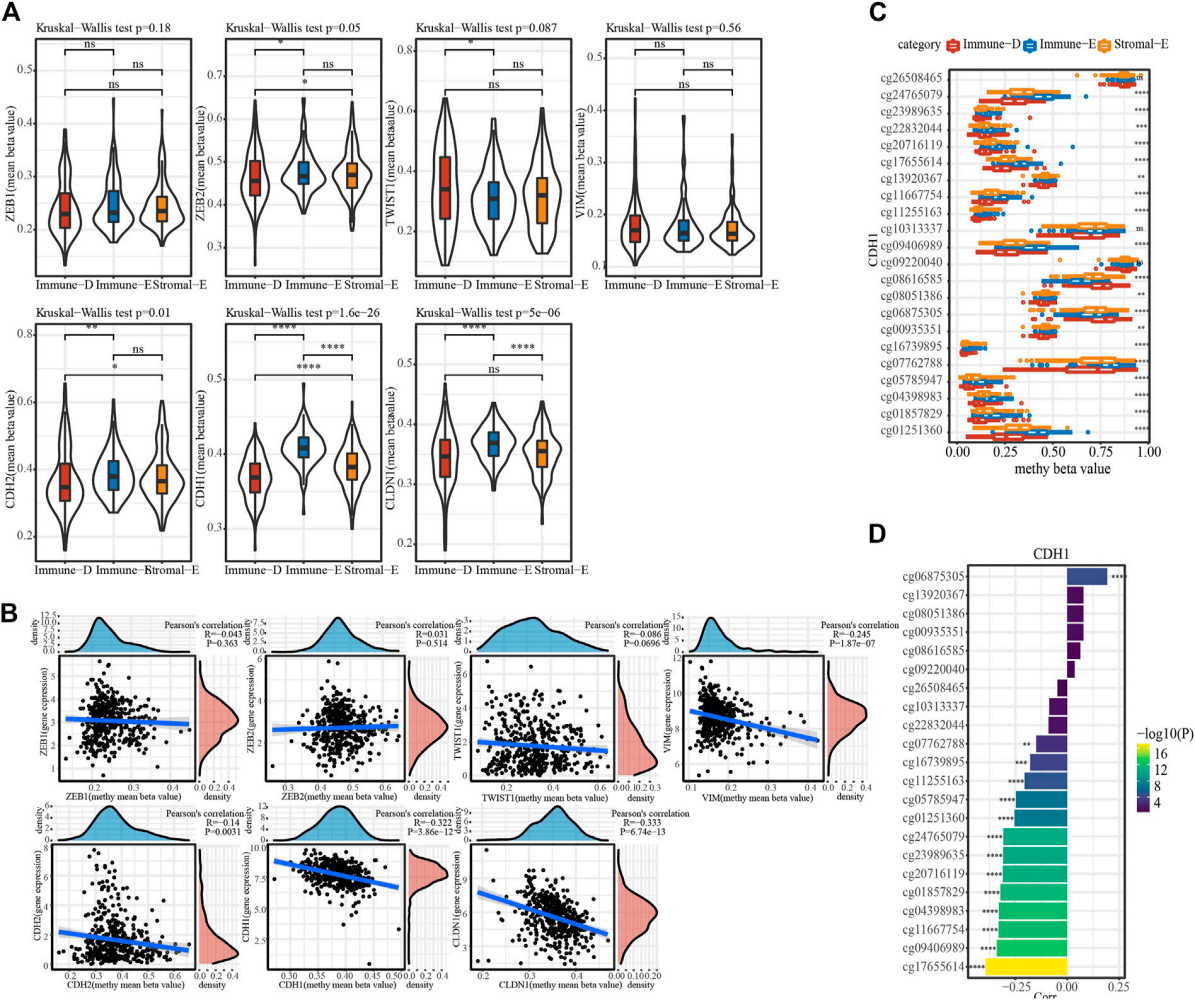
Methylation features of different subtypes. **(A)**, The distribution of methylation value of EMT-promoting gene in subtypes. **(B)**, Correlation analysis of methylation value and expression value of EMT-promoting gene. **(C)**, The beta value of cg probe site of gene CDH1 in subtype Differences in distribution among subtypes. **(D)**. Correlation of beta values of cg probe sites for CDH1 with CDH1 gene expression. Ns means no significance, * means *p-*value < 0.05, ** means *p-*value < 0.01, *** means *p-*value < 0.001, **** means *p-*value < 0.0001.

The methylation status of genes plays an important role in gene expression. DNA methylation analysis of seven EMT-promoting genes showed that the methylation status of ZEB1 and VIM did not differ among subtypes in TCGA dataset, while the methylation status of ZEB2, TWIST1, CDH2, CDH1 and CLDN1 were significantly different in each subtype (Figure 5A). The correlation analysis between gene expression value and methylation status showed that the gene expression values of VIM, CDH2, CDH1, and CLDN1 were significantly negatively correlated with methylation status (Figure 5B). The distribution of cg sites of CDH1 in different subtypes in TCGA dataset showed that some sites had significant differences in different subtype groups, and that the highest value in Immune-E was in a hypermethylated state (Figure 5C). From the correlation analysis of CDH1 cg loci and CDH1 gene expression, most of the cg loci and CDH1 gene expression showed a significant negative correlation (Figure 5D).

### 3.7 Immunotherapy and drug sensitivity of different subtypes

**FIGURE 6:**
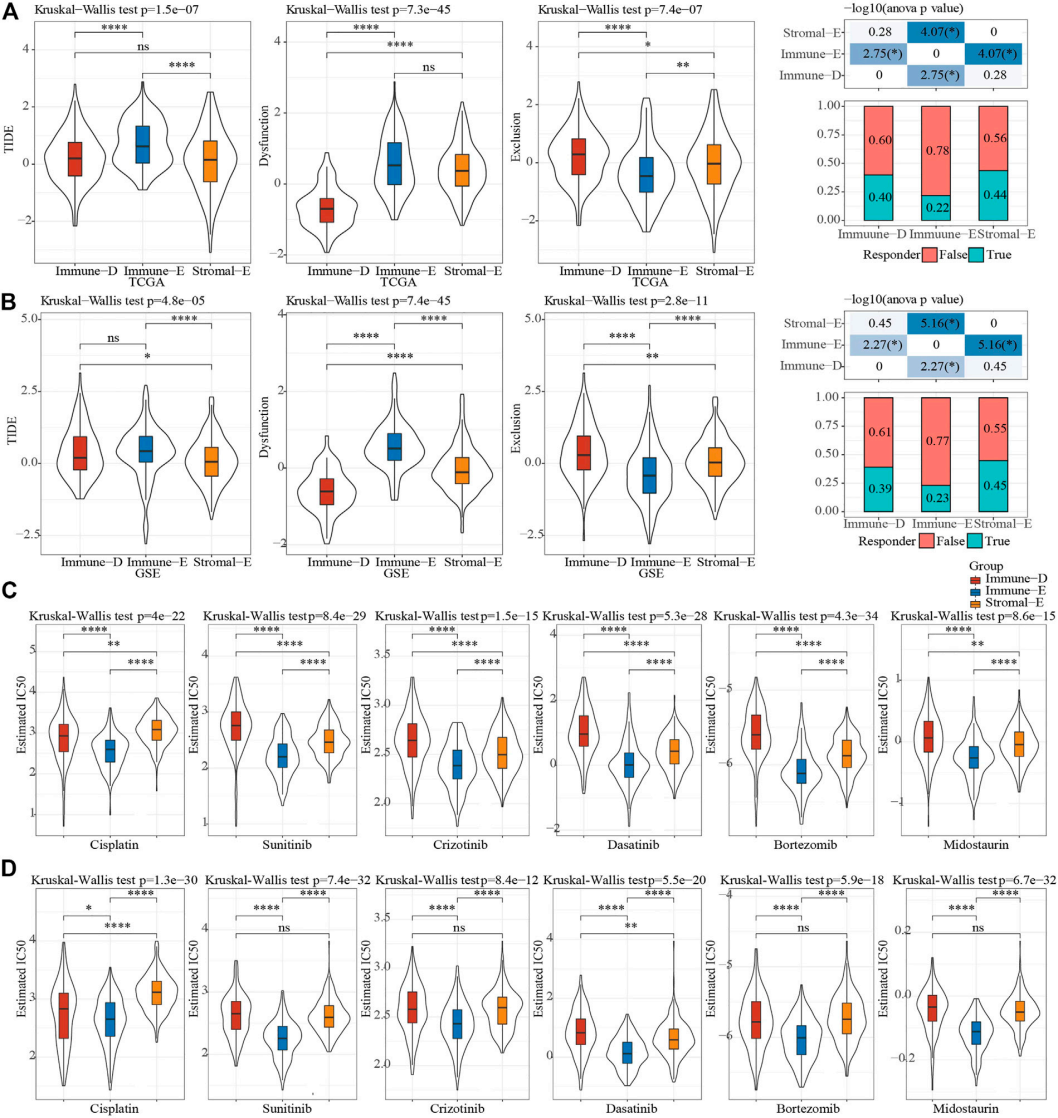
Sensitivity analysis of treatment options. **(A)**, Difference analysis of TIDE score of different immune subtypes in TCGA cohort. **(B)**, Difference analysis of TIDE score of different immune subtypes of GSE cohort. **(C)**, Difference analysis of drug IC50 of in TCGA cohort. **(D)**, Difference analysis of drug IC50 of in GSE cohort. Ns means no significance, * means *p-*value < 0.05, ** means *p-*value < 0.01, *** means *p-*value < 0.001, **** means *p-*value < 0.0001.

The TIDE (https://tide.dfci.harvard.edu/) algorithm was used to assess the potential clinical effects of immunotherapy on different molecular subtypes. In the TCGA cohort, the TIDE score of Immune-E was significantly higher than that of Stromal-E and Immune-D. The immunotherapy effect analysis indicated that the proportion of responses in Immune-E was only 22%, which was much lower than that of Stromal-E and Immune-D (Figure 6A). In GSE dataset, TIDE score was also higher in Immune-E. (Figure 6B). In addition, we also analyzed the response of different subtypes to the traditional chemotherapy drugs Cisplatin, Sunitinib, Crizotinib, Dasatinib, Bortezomib, Midostaurin. The results suggested that all the six drugs were more sensitive to the Immune-E subtype in both the TCGA and GSE cohorts (Figures 6C, D).

### 3.8 WGCNA to identify the key gene of subtypes

**FIGURE 7:**
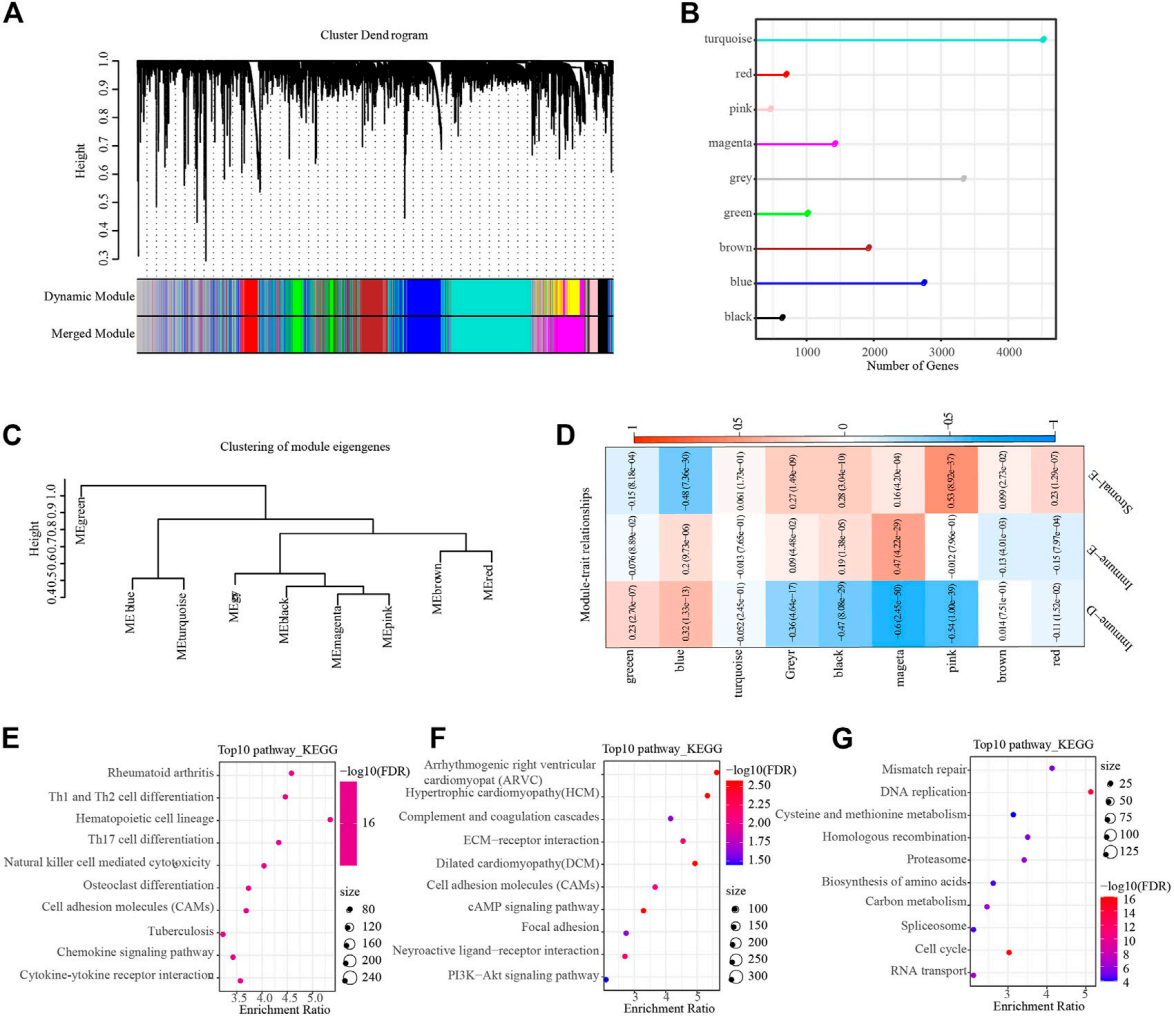
WGCNA identifies functional modules associated with different subtypes. **(A)**, Dendrogram of all genes clustered based on a dissimilarity measure. Different colors on the bottom panel represent different modules. **(B)**, Lollipop plot showing the number of genes in different modules. **(C)**, Clustering tree showing correlation between different modules. **(D)**, Heat map showing module correlation with different subtypes. **(E–G)**, The top 10 KEGG pathway in magenta (Immune-E), pink (Stromal-E) and blue (Immune-D) module, respectively.

Hierarchical cluster analysis of cohort samples showed no discrete samples in the TCGA cohorts (Supplementary Figure S3A). In order to ensure that the gene network we constructed conformed to the scale-free distribution, we set the β value to 9 at the time *R* ^2^ > 0.85 (Supplementary Figures S3A,S3C). After clustering the modules and merging the closer modules into a new module, we acquired a total of nine modules (Figure 7A). The lollipop graph showed the number of genes in each module. It can be observed that the turquoise color module had the largest number of genes, which was more than 4,000 genes (Figure 7B). The heat map of correlation analysis of each subtype and module showed that the Immune-D subtype had the highest positive correlation with the blue module, the Immune-E subtype had the highest positive correlation with the magenta module, and the Stromal-E subtype had the highest positive correlation with the pink module (Figures 7C,D). Further, we performed KEGG pathway analysis on the genes in the above three modules, and the results demonstrated that the genes in the magenta module were closely related to Natural killer cell mediated cytotoxicity, the genes in the pink module were closely related to ECM-receptor interaction, and the genes in the blue module were closely related to Genes were closely related to Mismatch repair, etc. (Figures 7E–G).

### 3.9 IMscore prognostic model construction

**FIGURE 8:**
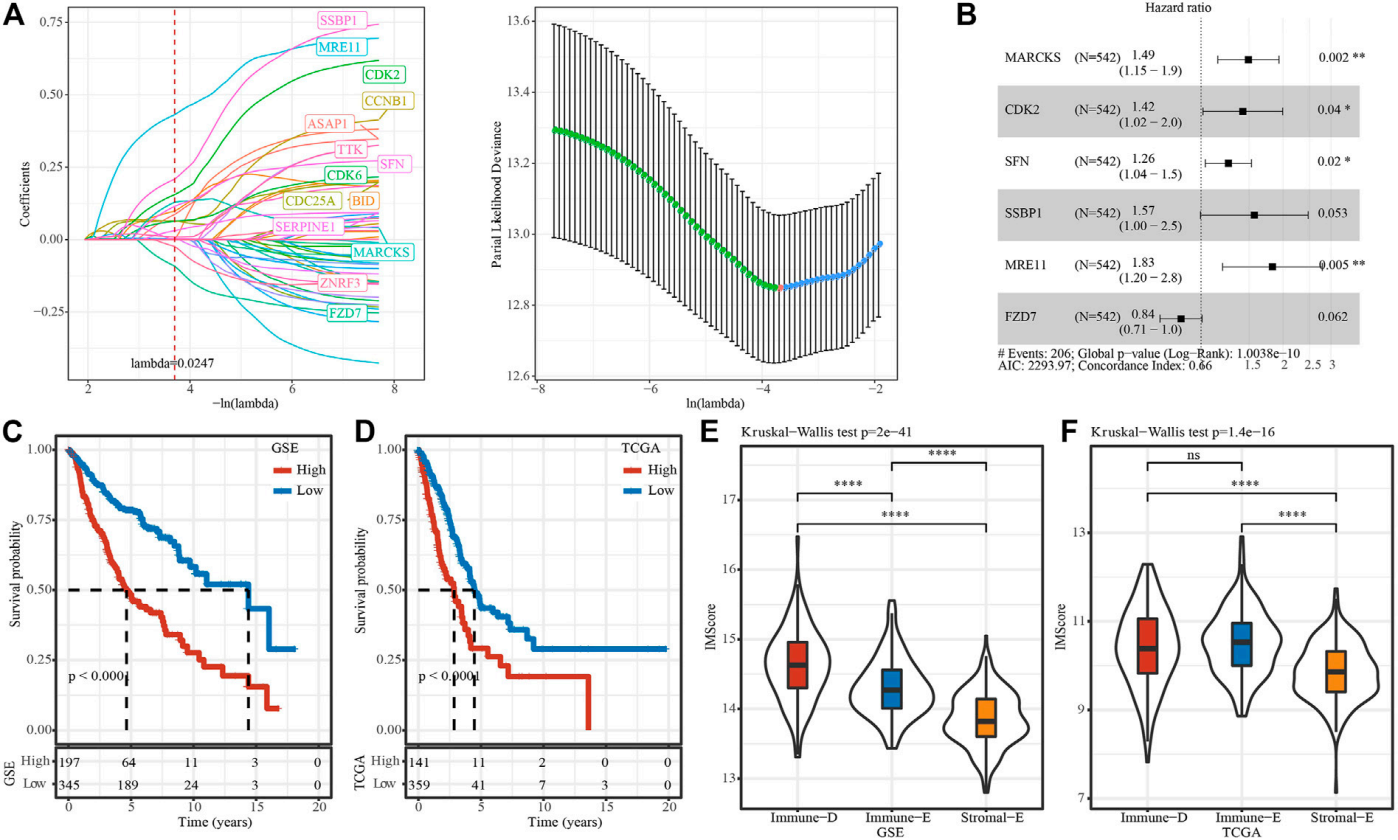
IMscore model to predict prognosis. **(A)**, Distribution of IMscore for each sample in the TCGA cohort. **(B)**, Distribution of LASSO coefficients for six genes. C and D, Kaplan-Meier survival analysis in different IMscore group in TCGA **(C)** and GSE **(D)** cohort. E and F, Differences in the distribution of IMscore among different subtypes (**(E)**, TCGA cohort; **(F)**, GSE cohort). Ns means no significance, * means *p-*value < 0.05, ** means *p-*value < 0.01, *** means *p-*value < 0.001, **** means *p-*value < 0.0001.

In GSE, six prognostic pathways (Fc gamma R-mediated phagocytosis mediated phagocytosis, p53 signaling, Mismatch repair, Homologous recombination, Wnt signaling, Cell cycle) from 15 pathways were identified by univariate COX analysis. Then, Pearson analysis on the genes in six pathways and six pathways score was used to select the top20 genes. Through univariate COX analysis and LASSO regression analysis, we obtained six genes form 20 genes as related genes affecting prognosis (Figures 8A, B), and the model score was developed based on the following formula: IMScore = 0.400*MARCKS + 0.353*CDK2 + 0.232*SFN + 0.450*SSBP1 + 0.604*MRE11 − 0.169*FZD7.

The survminer package was used to find the best cutoss of IMScore and divide the GSE and TCGA data set samples into high IMscore group (high group) and low IMscore group (low group). In the GSE cohort, survival analysis of high- and low-groups found that the prognosis of high groups was significantly worse than that of low groups. High group also had worse survival outcome in TCGA datset (Figures 8C, D). In addition, we compared the distribution differences of IMScore among subtypes in different datasets and found that: IMScore in Stromal-E had the lowest in GSE and TCGA, while Immune-D had the highest IMscore in GSE, Immune-E had highest IMscore in TCGA dataset (Figures 8E, F).

### 3.10 Prediction efficiency of IMscore prognostic model

The survival curve analysis of IMScore model in different cancer types showed that except ESCA, our IMScore had significant differences in high and low IMScore in all cancer types, and that the prognosis of high IMScore was significantly worse than that of low IMScore (Supplementary Figure S4). Furthermore, IMvigor210, GSE91061 and GSE135222 data were used to examine the efficiency of the IMscore model in immunotherapy. To evaluate the efficiency of IMscore, TIDE analysis was used as a control. For IMvigor210 cohort, survival analysis showed significant differences in survival among different groups (IMscore: *p-*value < 0.0001; TIDE, *p-*value = 0.012) (Supplementary Figures S5A, S5B). For GSE91061 cohort, survival analysis showed significant differences in survival among different IMscore groups (*p-*value < 0.023) (Supplementary Figures S5C). TIDE group was not statistically significant (*p-*value = 0.067) (Supplementary Figures S5D). For GSE135222 cohort, survival analysis showed no significant differences in IMscore or TIDE groups (IMscore: *p-*value = 0.055; TIDE, *p-*value = 0.051) (Supplementary Figures S5E, S5F). Notably, ROC curve analysis demonstrated that in the above three cohorts, the predictive efficiency of IMscore was higher than that of TIDE.

## 4 Conclusion

LUAD is one of the most common malignant tumors, with high metastasis rate and strong invasiveness. Its low 5-year survival rate seriously threatens the life and health of human beings (Charloux et al., 1997; Tan et al., 2016). Significantly, early-stage LUAD has been reported to be associated with a higher risk of postoperative recurrence and death (Bittner et al., 2014). The immune system has been shown to play a critical role and even determine different stages of cancer development and progression (Shurin, 2018). Hence, an accurate classification of LUAD patients according to immune characteristics and the identification of LUAD biomarkers have positive significance for the selection of LUAD treatment methods. A previous study reported six subtypes in LUAD, including Wound Healing, IFN-γ Dominant, Inflammatory, Lymphocyte Depleted, Immunologically Quiet, and TGF-β Dominant (Thorsson et al., 2018). However, in this research, three molecular subtypes classified by immunological features were obtained and defined as Immune-E, Stromal-E and Immune-D.

Kaplan-Meier survival analysis was able to determine survival differences between different subtypes (Goel et al., 2010). We found that Stromal-E subtype had a better prognosis and Immune-D had a worse prognosis. To gain a deeper understanding of the differences in survival between the different subtypes, we used *Estimate* software for immune calculating index scores and tumor purity scores (Yoshihara et al., 2013). The results showed that higher tumor purity and lower immune cell scores were in the Immune-D subtype, which accounted for the poorer prognosis of the subtype.

Recently, immunotherapy has also been developed as a new treatment for LUAD (Saito et al., 2018). Moreover, antibodies against PD-1 and PD-L1 have been reported to be effective in the treatment of various malignancies (Antonia et al., 2017; Bie et al., 2021). Although the biology of the TIME driving these responses was not fully understood, it is critical for the design of immunotherapeutic strategies. We found that the expression level of PD1, PD-L1, CTLA4 and LAG3 was higher in Immune-E subtype. However, as we mentioned above, the survival analysis showed that the Stromal-E subtype had a better prognosis, which suggested the complexity of tumorigenesis mechanisms.

TMB is an emerging tumor biomarker and it is associated with response to PD-1/PD-L1 targeted therapies in lung cancer (Spigel et al., 2016). We found that TMB in the Stromal-E subtype was significantly lower than in the Immune-E and Immune-D subtypes. TP53, known as the guardian of the genome, is one of the most well-known tumor suppressor genes (Surget et al., 2014). Interestingly, the mutation analysis suggested that Stromal-E subtype had a lower TP53 mutation rate of only 32%, while the other two subtypes both exceeded 50%, which explained a better prognosis of Stromal-E subtype. Moreover, high levels of TIDE scores suggested that Immune-E was more likely to occur immune escape, suggesting that the Immune-E subtype had limited benefit from immunotherapy (Jiang et al., 2018). Interestingly, although the Immune-E subtype had little limited benefit from immunotherapy, IC50 analysis of chemotherapeutic agents showed that this subtype was more sensitive to chemotherapeutic agents, including Cisplatin, Sunitinib, Crizotinib, Dasatinib, Bortezomib and Midostaurin.

The above analysis showed a high heterogeneity among different immune subtypes. In order to better understand the differences between different subtypes, we used WGCNA analysis to identify hub genes among each subtype. Extensive analyses showed that WGCNA was an effective method for identifying phenotype-genotype linkages and biomarkers and therapeutic targets (Niemira et al., 2019; Ma et al., 2021; Zhang et al., 2022). Nine modules were obtained, and pink module was highly associated with Stromal-E subtype. Further function enrichment analysis showed that the hub genes in pink module were involved in ECM-receptor interaction pathway. Tumor progression depends not only on cell-autonomous changes in tumor cells, but also on the changes within the microenvironment (Götte and Kovalszky, 2018). An important feature of the dysregulated lung cancer microenvironment is the altered extracellular matrix (ECM), which can promote tumor angiogenesis, allow tumor cell immune escape, etc. (Mahale et al., 2016). In addition, magenta module was positively correlated with Immune-E subtype and participated in natural killer cell mediated cytotoxicity.

Notably, we developed an IMscore model containing six immune-related genes (MARCKS, CDK2, SFN, SSBP1, MRE11 and FZD7) to predict LUAD prognosis. CCNA2-CDK2 complex have been reported to inhibit LUAD progression (Li et al., 2021). CDK2 also was a biomarker for other cancers and next-generation CDK2 inhibitors play an increasingly pivotal role in the treatment of cancer (Wadler, 2001; Chohan et al., 2015; Tadesse et al., 2020). SFN gene encodes a protein participating in regulating epithelial-mesenchymal interaction (Asdaghi et al., 2012). Aya Shiba-Ishii reported that SFN promoted early progression of LUAD by activating cell proliferation (Shiba‐Ishii, 2021). SSBP1, MRE11 and FZD7 have been considered as potential treatment sites of LUAD (Sun et al., 2017; Wang et al., 2017; Zeng et al., 2020). Kaplan-Meier survival analysis showed the high IMScore group had a significantly lower prognosis than the low IMScore group. Then, we detected the IMScore in three immune-related subtypes. As expected, the lowest score of IMScore in Stromal-E subtype was obtained. To explore the efficiency of the IMscore model, we used the expression profile data of the remaining 32 cancer types in the TCGA database for validation, and found that IMScore was significantly different in all cancer types except ESCA.

## 5 Conclusion

In this study, we used immune-related signaling pathways to classify LUAD and obtained three different subtypes, which had great differences in survival, gene mutation, CNV, gene methylation, etc. We further constructed an IMscore model to predict the prognosis among different subtypes of LUAD, and observed that the IMscore model had a higher efficiency. In addition, data in different cancers further confirmed the validity of the IMscore model.

## Data Availability

The original contributions presented in the study are included in the article/Supplementary Material, further inquiries can be directed to the corresponding author.
