# Supplementary figures and images for: Characterization of lung adenocarcinoma based on immunophenotyping and constructing an immune scoring model to predict prognosis
Source: Front Pharmacol. 2022 Dec 19;13:1081244. doi: 10.3389/fphar.2022.1081244 (PMC9806149; doi:10.3389/fphar.2022.1081244)

A

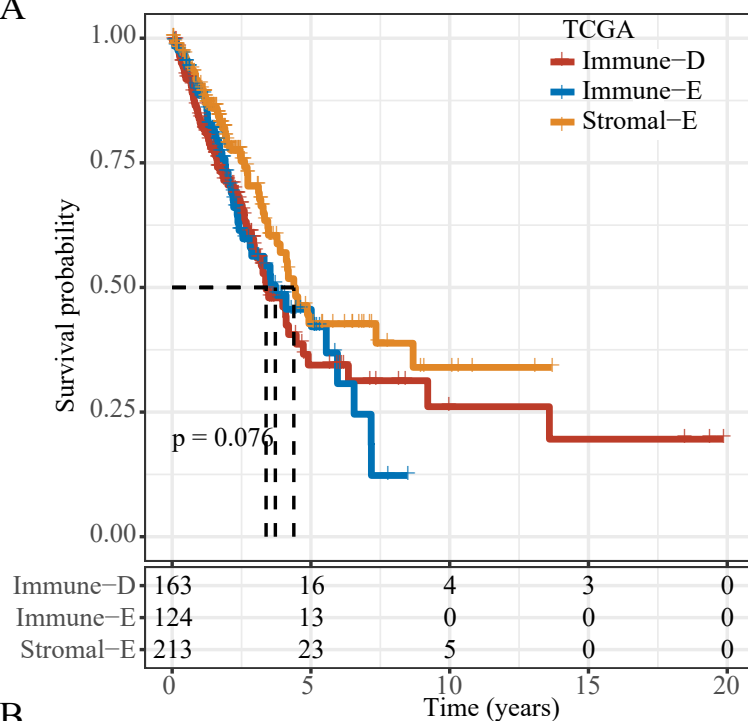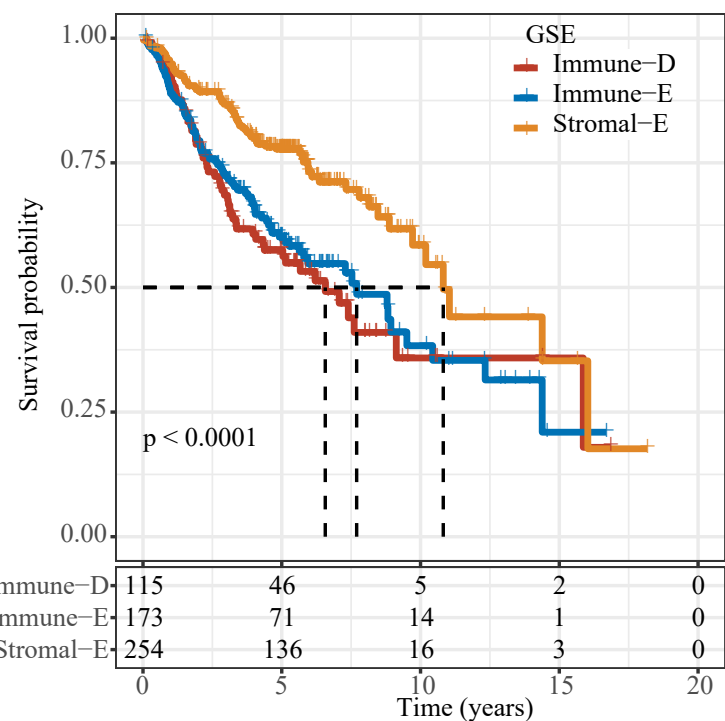

B

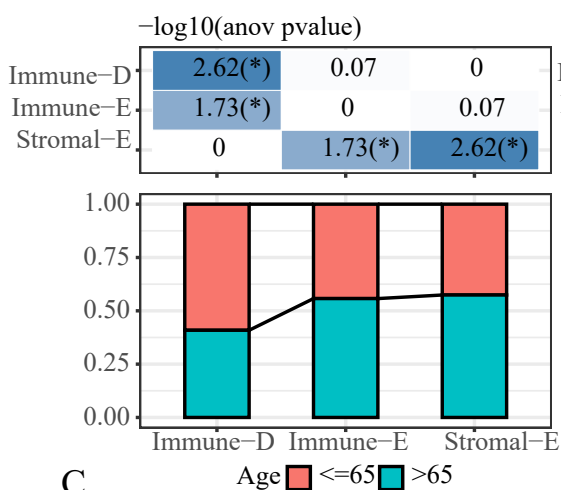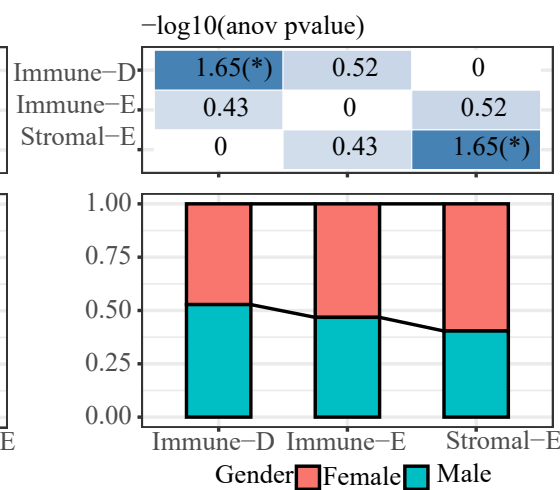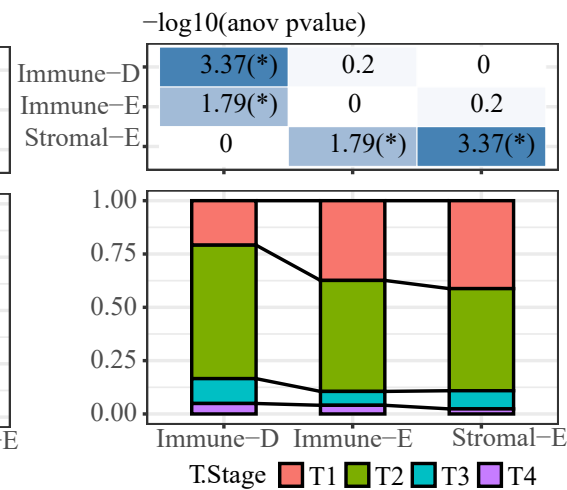

C

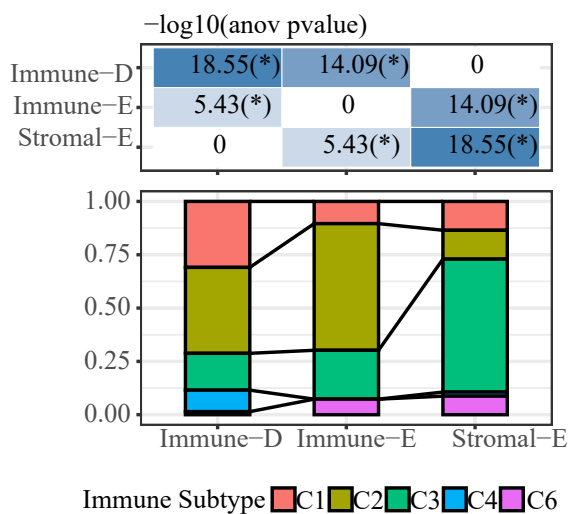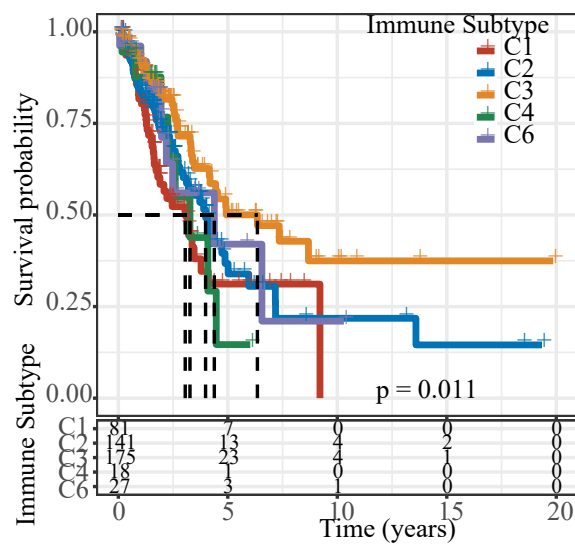

Supplement: Supplementary file 1 [file DataSheet2.PDF]

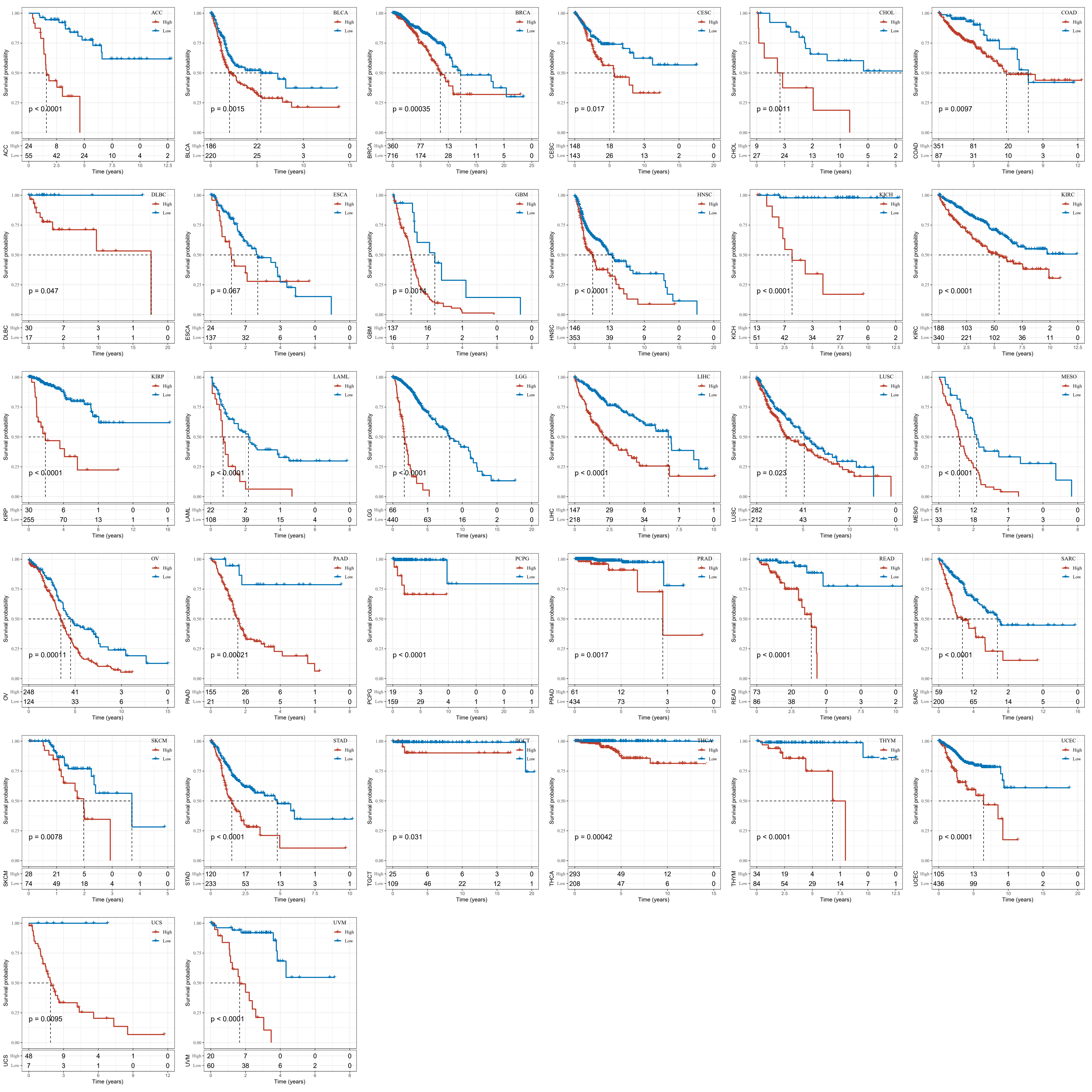

Supplement: Supplementary file 2 [file DataSheet4.PDF]

A

## Sample clustering to detect outliers

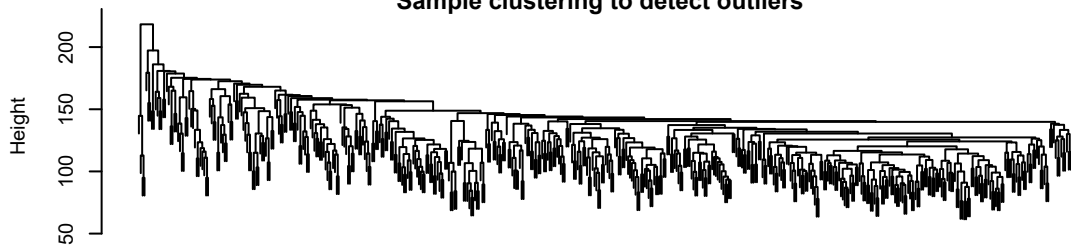

B

## Scale independence

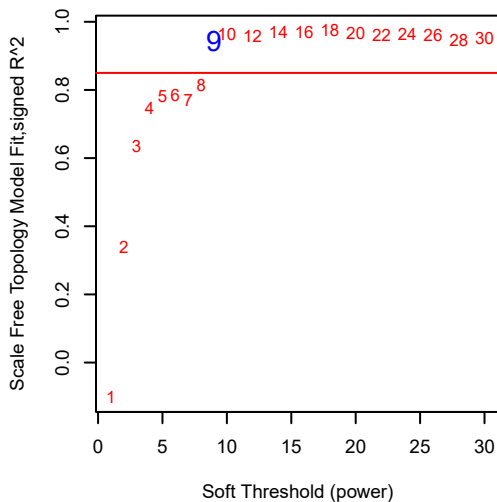

C

## Mean connectivity

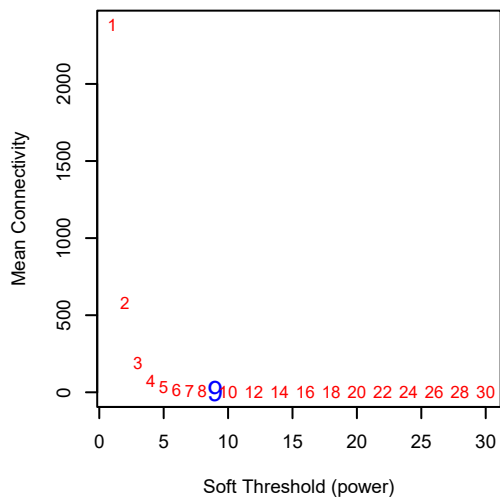

Supplement: Supplementary file 4 [file DataSheet3.PDF]

**A**

groups — GSE30219 — GSE31210 — GSE37745 — GSE50081

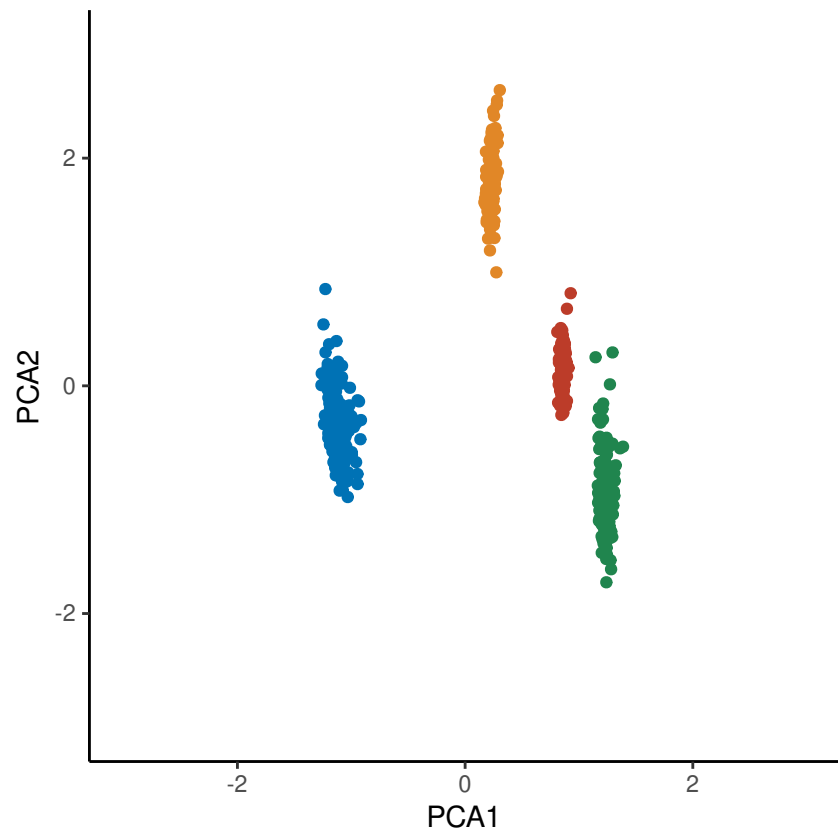**B**

groups — GSE30219 — GSE31210 — GSE37745 — GSE50081

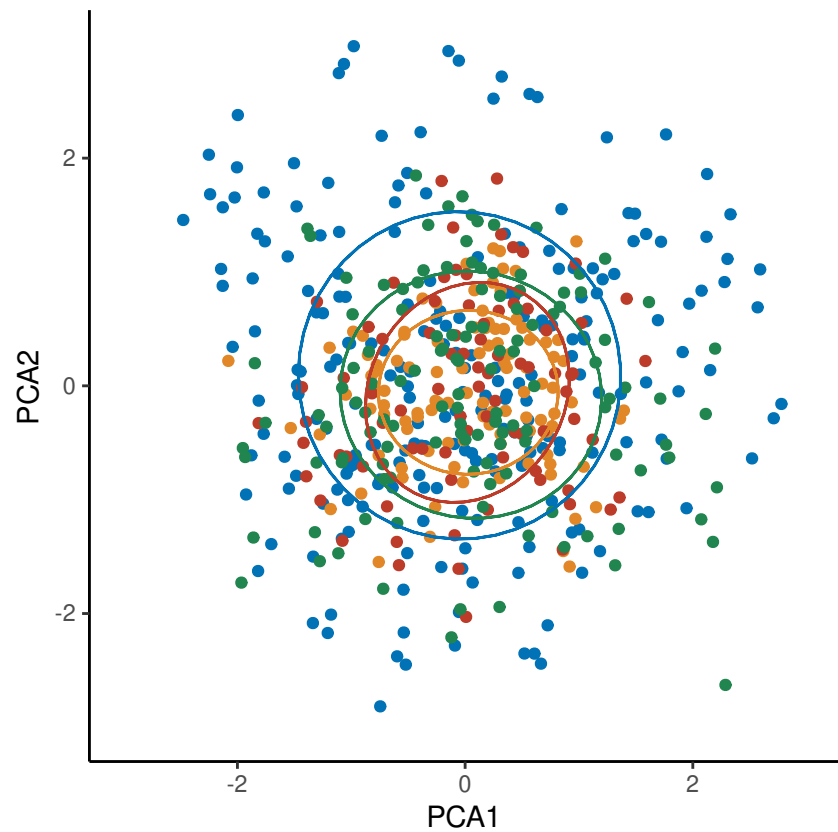

Supplement: Supplementary file 5 [file DataSheet1.PDF]

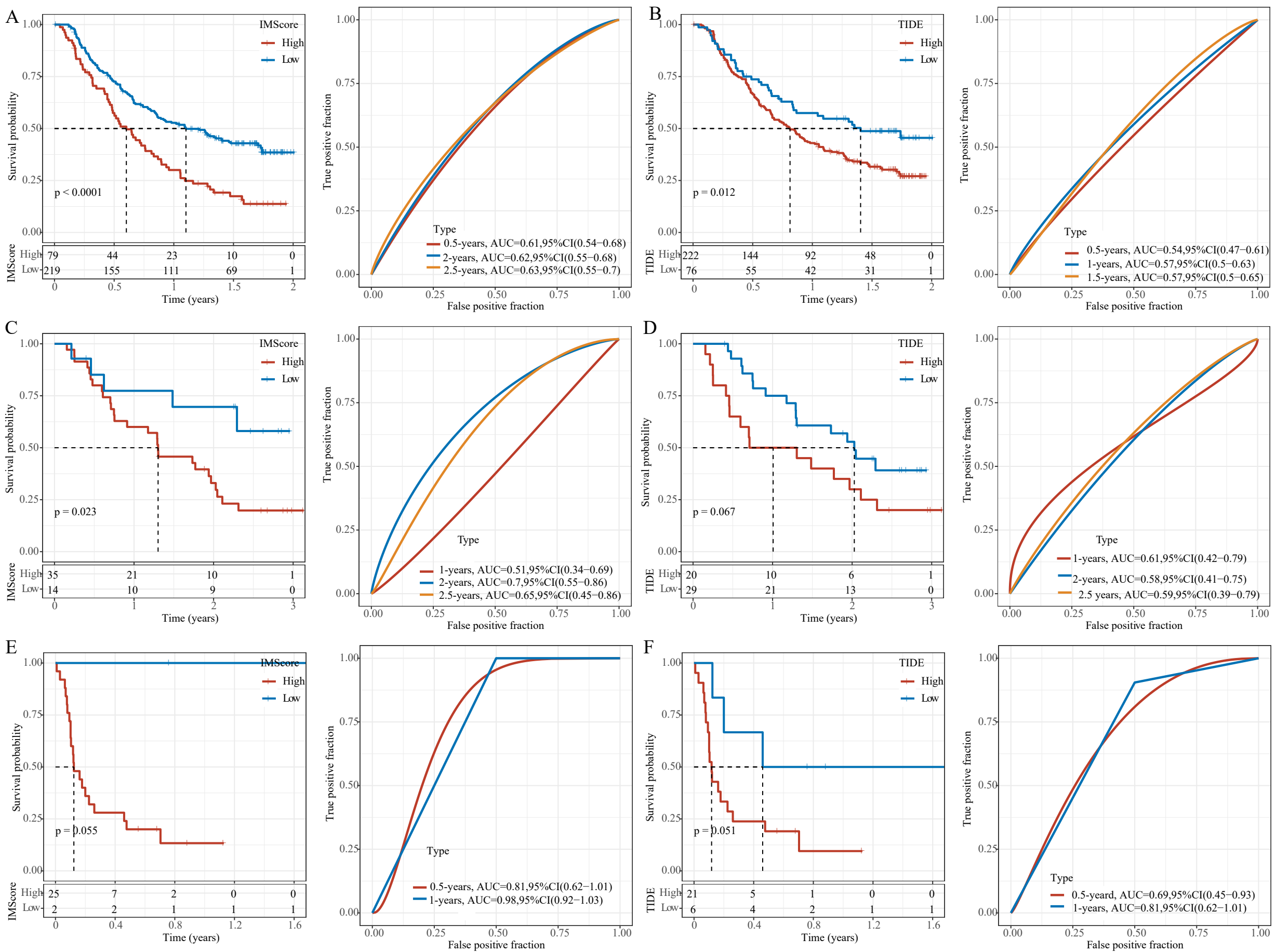

Supplement: Supplementary file 6 [file DataSheet5.PDF]
