# Supplementary material for: Characterization of lung adenocarcinoma based on immunophenotyping and constructing an immune scoring model to predict prognosis
Source: Front Pharmacol. 2022 Dec 19;13:1081244. doi: 10.3389/fphar.2022.1081244 (PMC9806149; doi:10.3389/fphar.2022.1081244)
Supplement: Supplementary file 3 [file DataSheet6.docx]

**Supplementary figure legends**

**Fig S1.** **Removal of batch effects.** A, PCA distribution before removing batch effects. B, PCA distribution after removing batch effects.

**Fig S2. Clinical features of different subtypes.** A, Kaplan-Meier survival analysis of different subtypes in TCGA (left) and GSE (right) cohorts. B, Comparative analysis of different clinical features in different subtypes, followed by Age, Gender and T Stage. C, Comparison of distribution subtypes in this study with those published in previous studies. Type comparison on the left, Kaplan-Meier survival analysis on the right. Ns means no significance, * means *pvalue* < 0.05, ** means *pvalue* < 0.01, *** means *pvalue* < 0.001, **** means *pvalue* < 0.0001.

**Fig S3. WGCNA analysis supporting for Fig 8.** A, Hierarchical cluster analysis of all cohort data. B, The scale-free fit index distribution and red line means the threshold. C, The mean connectivity of different scale-free fit index.

**Fig S4. Kaplan-Meier survival analysis supporting for Fig 10.** Kaplan-Meier survival analysis in different IMscore group in different cancers.

**Fig S5. IMscore model efficiency in different data.** A, IMscore survival curve and ROC curve in IMvigor210. B, TIDE survival curve and ROC curve in IMvigor210. C, IMscore survival curve and ROC curve in GSE91061 cohort. D, TIDE survival curve and ROC curve in GSE91061 cohort. E, IMscore survival curve and ROC curve in GSE135222 cohort. F, TIDE survival curve and ROC curve in GSE135222 cohort.

**Table S1.** immune pathways
